# The timing of growth faltering has important implications for observational analyses of the underlying determinants of nutrition outcomes

**DOI:** 10.1371/journal.pone.0195904

**Published:** 2018-04-25

**Authors:** Harold Alderman, Derek Headey

**Affiliations:** International Food Policy Research Institute, Washington, District of Columbia, United States of America; The Hospital for Sick Children, CANADA

## Abstract

**Background:**

Growth faltering largely occurs in the first 23 months after birth and is thought to be largely determined by various harmful or protective socioeconomic conditions. Children 23 months or younger, however, have only been partially exposed to these conditions, implying that statistical associations between these conditions and child growth may be substantially smaller in samples that include younger children.

**Objectives:**

To test the prediction that associations between child anthropometric outcomes and various socioeconomic conditions are systematically different for older and younger children.

**Methods:**

We analyzed data for 699,421 children aged 0–59 months, drawn from 125 DHS implemented between 1992 and 2014 in 57 countries. The outcome variables were height-for-age Z scores (HAZ) and stunting (HAZ<-2), and weight-for-height z scores (WHZ) and wasting (WHZ<-2). Independent variables included household wealth, parental education, maternal height, demographic factors, and exposure to WASH and health services. We used age-disaggregated regressions to examine how the associations between dependent and independent variables vary across different child age ranges.

**Results:**

Non-parametric regression results reaffirmed that most linear growth faltering and wasting takes place prior to 23 months of age. Estimates of the magnitude of association with wealth, education and improved toilet use from HAZ regressions are systematically larger in the sample of children 24–59 months than in the 0–23 month or 0–59 month samples; the reverse is true for WHZ regressions.

**Conclusions:**

Previous observational analyses appear to substantially underestimate the protective impacts of a wide range of underlying determinants on stunting. Conversely, wasting rates are typically low for children 24–59 months, implying that associations between underlying conditions and wasting may be stronger for children 0–23 months of age.

Such analyses should pay closer attention to age disaggregation; researchers should be aware of the age effect reported in the current study and present analysis stratified by age.

## Introduction

Influential research by Victora et al. and Shrimpton et al. [1, 2] graphically demonstrated that growth faltering using the commonly employed metric of height-for-age Z scores (HAZ) among young children in developing countries largely occurs prior to a child’s second birthday. Although growth faltering can continue beyond 24 months, which is obscured by using HAZ in these analyses [3], there is little doubt that infants and young children are exceptionally vulnerable to poor diets and infection during these first 1000 days. However, there is less consensus on the importance of basic causes such as socio-economic and environmental factors that indirectly influence underlying determinants of stunting and wasting, with significant bodies of research assessing, re-assessing and debating the magnitude and relative roles of wealth, income and economic growth [4–7], parental education [8–10], household and community sanitation [11–13] and demographic factors [14, 15]. Other studies more agnostically attempt to identify which of these underlying determinants explain reductions in stunting or wasting over time in nutrition success stories [16–19]. The relative scarcity of panel surveys on child nutrition means that relatively few studies use longitudinal data to ascertain the role of underlying determinants of undernutrition [20, 21]. However, countless other studies explore the cross-sectional determinants of stunting using single surveys, including several hundred studies using Demographic Health Survey (DHS) data on child nutrition [22, 23].

With very few exceptions, however, these observational analyses mostly focus on linear growth measures for children 0–59 months of age, or in some cases 0–36 months of age [6]. By including sub-samples of younger children (i.e. 0–23 months) still in the process of growth faltering, these studies fail to fully take into account the implication of the timing of growth faltering on the association between child growth and its various determinants. Specifically, in standard multivariable regression models the coefficients on the various underlying determinants of nutrition can be thought of as a weighted average of the associations that exist across the full age range being used. However, children aged 0–23 months should arguably be analyzed separately from older children, as their nutritional status does not reflect the full impacts of various postnatal nutritional insults, or conversely, the benefits of various postnatal protective factors (e.g. wealth, education, sanitation) have not manifested fully. This would imply that regression analyses of linear growth that incorporate children 0–23 months will produce attenuated coefficients on these protective factors.

We term this attenuation “partial exposure bias”. This bias has been recognized in the experimental literature on nutrition with reference to initiating treatments to children from appropriately young age groups (i.e. less than 24 months), and assessing impacts when children have passed 24 months of age [24, 25]. For example, a recent sanitation trial explicitly tested sensitivity of their core results to age of first exposure to the program [26], although previous sanitation trials that only focus on stunting as a secondary outcome indicator tended to ignore issues of exposure bias [27]. Exposure bias has also been recognized in several observational studies of the underlying determinants of nutrition [8, 17, 28], though the extent of this bias has not been extensively quantified. Partial exposure bias may result from studies that fail to account for the cumulative impact of an underlying determinant of nutrition when that factor has an influence that is important over many months. It may also be an issue when the determinant has an age specific role that is biologically determined.

With weight-for-height Z-scores (WHZ) or wasting, one might expect opposite patterns with attenuation bias stemming from the inclusion of older children (i.e. 24–59 months). In most regions, WHZ scores decline from birth and reach a modest nadir at around 12 months, but improve somewhat thereafter; in part, this is consistent with older children gradually attaining stronger immune systems that diminish the impact of infections on a child’s weight [1, 2]. As a result, lower levels of wasting among slightly older children (24-59m) likely lead to attenuated regression coefficients on most underlying determinants because there is less variation in WHZ scores to explain (a statistical explanation) and because of greater immunological robustness to insults that might have had more adverse impacts at earlier stages of life (a biological reason) [29].

In this paper our objectives are twofold. First, we aim to identify whether associations between HAZ/stunting and their basic nutritional determinants are significantly attenuated when using younger samples of children (e.g. 0.23 months) relative to older samples of children (24–59 months). Our second objective is to identify whether there is any partial exposure bias in regressions exploring associations of WHZ/wasting for children aged 0–59 months relative to samples of younger children (0–23 months). In both cases evidence of attenuation would imply that the conventional approach in observational analyses of nutrition, using the full sample of children 0–59 months of age in observational regression analyses, leads to underestimation of the potential contribution of various basic determinants (e.g. wealth, parental education, sanitation) to reducing child stunting or wasting.

## Methods

We analyze all suitable DHS surveys [30] to broadly replicate other recent multi-country DHS studies on the underlying determinants of nutrition. We excluded DHS surveys that did not measure HAZ or WHZ scores (relative to 2007 WHO growth standards for the entirety of the 0–59 month age range, as well as observations with Z scores below -6 or above 6. We also excluded surveys that did not collect data on correlates of nutrition that are commonly used in analysis of DHS data, such as a household wealth index, parental education, maternal nutrition status (height), sanitation and water source types, birth spacing, number of children ever born, and whether a child was born at home or in a medical facility. One point of note is that we construct our own wealth index using four indicators of housing characteristics and four household assets using the conventional approach of deriving index weights from principal components analysis [31]. In this case, however, we derived an index with weights common to all countries to improve comparability across countries. We note, however, that this index is very highly correlated with an index that uses country-specific weights (r = 0.97 across all countries).

The final data set contains a sample of 125 DHS surveys with data on 699,421 children from 57 countries. Countries, survey years, and age-specific stunting and wasting rates are listed in S1 Table. Just over half (54%) of these observations pertain to children from sub-Saharan Africa, 9% from South Asia, 10% from the Middle East and North Africa, 22% from Latin America and the Caribbean, 3% from Eastern Europe and Central Asia and just 2% from East Asia. Hence the sample is suitably comprised of lower income countries with high rates of undernutrition. However, we do not apply population weights in any of our analyses, though our results are robust to the use of weights.

To explore evidence of partial exposure bias, we used STATA v14 to implement three complementary statistical approaches. As a first step, we verified that the age patterns in the recent data are true to type as in Victora et al. [1] and Shrimpton et al. [2]. Following recent studies [16, 17], we regressed HAZ and WHZ scores against child age using a local polynomial regression (the *lpolyci* command in STATA v14) and plotted the smoothed curve with 95% confidence intervals. These plots are similar to the conventional growth faltering curves reported in earlier papers, with the main difference being somewhat smoother regression-based plots which treat the data as stochastic. This is desirable if there are errors in the reporting of children’s height or ages, as a recent study suggests [32].

As a second step we estimate least squares regressions of HAZ and WHZ for the full sample of children 0–59 months as well as separately for children 0–23 months and 24–59 months. We then compare the percentage differences in coefficients generated by the different samples to look for evidence of partial exposure bias, and formally test the null hypothesis of coefficient equality across samples using Wald tests. Finally, we divide the sample even further into smaller age brackets (0–5 months, 6–11 months, 12–17 months, 18–23 months, 24–35 months, 26–47 months and 48–59 months), re-estimate least squares regression models, and plot the coefficients for each sub-sample using the *coefplot* command in STATA v14 with 95% confidence intervals. This allows us to examine potentially more complex coefficient patterns which can be compared to the progressions of HAZ and WHZ by child age estimated in step 1. The standard errors in Steps 2 and 3 are adjusted for survey clustering (though this is not possible with the nonparametric regressions in Step 1).

## Results

### Sample characteristics

Table 1 reports the means and standard deviations of the variables included in the analysis. As expected given the settings for DHS data, this sample of children has low mean HAZ scores (-1.42), and their corresponding households mostly have limited access to education, health care, sanitation and improved water sources. Fertility rates are also high on average, as is teenage motherhood and short stature among mothers. Around two-thirds of the sample are rural, and 51% are boys.

**Table 1:** Descriptive statistics for a sample of 125 Demographic Health Surveys from 57 countries.

Variable	Mean	Std. Dev.
HAZ	-1.43	1.70
WHZ	-0.05	1.52
Poorest tercile (= 1)	0.38	0.49
Middle tercile (= 1)	0.29	0.45
Highest tercile (= 1)	0.33	0.47
Women >9years education (= 1)	0.25	0.44
Partner >9years education (= 1)	0.33	0.47
Partner missing (= 1)	0.06	0.23
Born at home (= 1)	0.45	0.50
Short birth interval (= 1)	0.15	0.36
3–4 children (= 1)	0.31	0.46
5-plus children (= 1)	0.31	0.46
Teenage mother (<20 years)	0.17	0.38
Woman <145cm (= 1)	0.05	0.23
Woman 145-150cm (= 1)	0.14	0.34
Woman 150-155cm (= 1)	0.24	0.43
Improved latrine (= 1)	0.26	0.44
Basic latrine (= 1)	0.45	0.50
Improved water (= 1)	0.65	0.48
Rural (= 1)	0.65	0.48
Boy (= 1)	0.51	0.50
Child age (months)	28.91	17.09

Source: Statistics derived from 125 Demographic Health Surveys from 57 countries. (N = 669,421).

**Fig 1:**
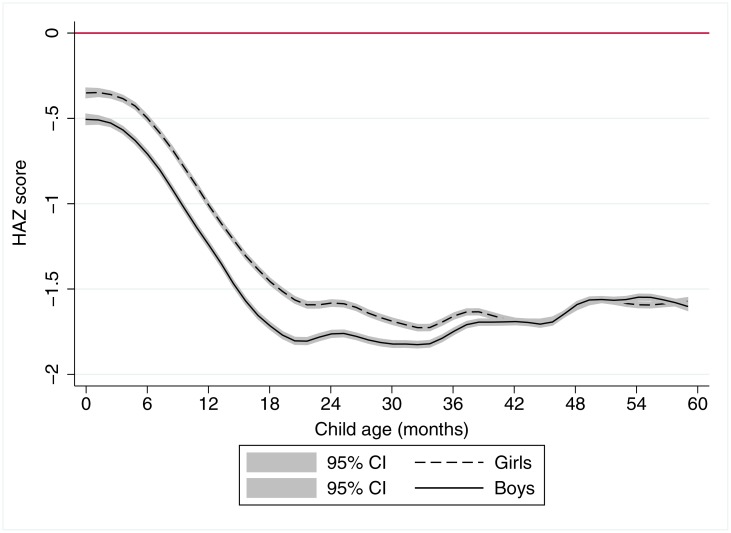
A local polynomial regression plot of height-for-age z score against child age for 699,421 children aged 0–57 months in 58 countries. Notes: The graph is based on local polynomial smoothing estimates of HAZ scores against child age for 699,421 children from 125 Demographic Health Surveys for 57 countries. 95% confidence intervals (CI) are reported in grey shading.

Fig 1 reaffirms the rapid decline of stature for age relative to international norms from birth until approximately age 21 months. Thereafter, there is no further decline in HAZ scores, confirming that growth faltering as measured by HAZ largely takes place in the first 1000 days of life. However, we do not plot the data by region as was reported in Victora et al. [1], though we do present the patterns by gender. This shows that boys tend to be born smaller than girls relative to the gender-specific international norms, and to remain substantially below these norms throughout the first 1000 days. Thereafter, however, the apparent advantage that girls in low income setting have with respect to boys gradually dissipates and disappears altogether by age 40 months. This gender difference in HAZ scores by a child’s age has occasionally been reported in the literature for individual countries [33], but is not often presented as a global pattern. Another point of note is that, in keeping with the graphical presentation, S1 Table confirms that stunting levels are substantially higher among children 24–59 months than children 0–23 months.

**Fig 2:**
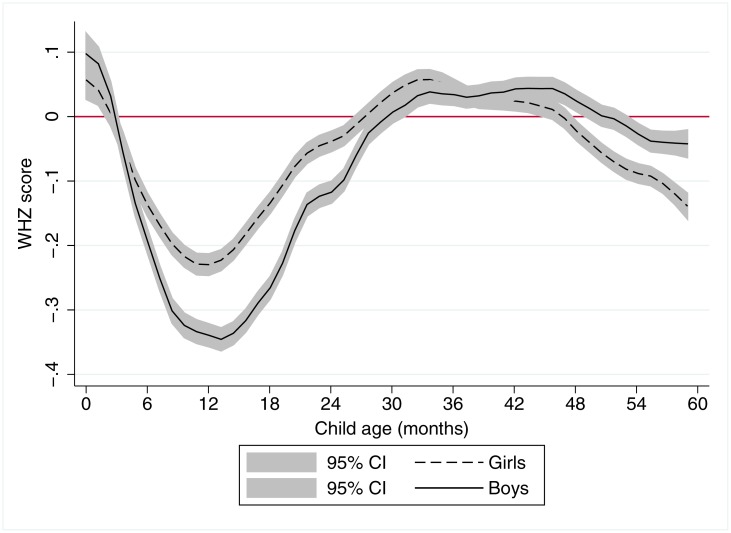
A local polynomial regression plot of weight-for-height z score against child age for 699,421 children aged 0–59 months in 57 countries. Notes: The graph is based on local polynomial smoothing estimates of WHZ scores against child age for 699,421 children from 125 Demographic Health Surveys for 57 countries. 95% confidence intervals (CI) are reported in grey shading.

Fig 2 also reaffirms earlier results for WHZ scores, which are strikingly different to HAZ dynamics. On average, both boys and girls in developing countries appear to be born with adequate WHZ, but they quickly fall negative, with a nadir at roughly 12–13 months of age (South Asia is an exception since WHZ is low from birth in this region, although there is also a further decline in the first two years). After 12–13 months mean WHZ scores recover to the mean of the international reference population by around 26 months and remain stable to around 48 months before again declining slightly. Consistent with Fig 1, mean WHZ scores for boys are well below those of girls in the first 24 months of postnatal life, though from approximately 44 to 59 months girls have significantly lower mean WHZ scores than boys S1 Table confirms that wasting rates are generally higher among children 0–23 months than among children 24–59 months.

### Regression analysis

Table 2 reports the multivariable regression results from Step 2 of our analysis, HAZ regressions for the full sample of children 0–59 months, and the 0–23 month and 24–59 month subsamples. With the exception of the father being present, all coefficients are statistically significant in the overall regressions as well as in all but one of the age subsamples. However, the magnitudes on the coefficients often differ across samples; consistent with the associations of household resources increasing over time, the coefficients of many of the variables are much larger in absolute magnitude for the sample of children 24 months and older compared to younger children. Moreover, these differences are typically statistically significant at the 5% level or higher. For example, the coefficients on parental education and household wealth terciles increased by 30–62% when switching from the 0–23 month to the 24–59 month sample. In comparison to the conventional 0–59 month sample, the coefficients on wealth and education in the 24–59 month sample are 8–24% larger. Similar indications of partial exposure bias are evident in the coefficients for most other variables too, with the main exception being maternal height, which is unaffected by age restrictions, presumably because maternal height influences size at birth, and thereby sets the trajectory for postnatal growth. Overall, the results strongly suggest that cross sectional regressions for the conventional 0–59 month sample substantially underestimates the relationship between children’s growth outcomes and key underlying basic determinants.

**Table 2:** Least squares regressions of child HAZ against standard explanatory variables for children aged 0–59 months, 0–23 months and 24–59 months from 57 developing countries.

		(1)	(2)	(3)	Differences across samples:^a^	
		N = 699,421	N = 288,754	N = 410,667	(3) minus (1)	(3) minus (2)
		0–59 months	0–23 months	24–59 months		
Variable						
(base category)						
Middle wealth tercile	coef.	0.141***	0.109***	0.164***	15.3%***	46.9%***
(vs lowest)	p-val	<0.001	<0.001	<0.001		
Upper wealth tercile	coef.	0.372***	0.289***	0.426***	14.7%***	47.3%***
(vs lowest)	p-val	<0.001	<0.001	<0.001		
Mother 9 years school	coef.	0.161***	0.120***	0.202***	24.1%***	62.0%***
(vs none)	p-val	<0.001	<0.001	<0.001		
Father 9 years school	coef.	0.109***	0.094***	0.121***	10.7%***	30.5%***
(vs none)	p-val	<0.001	<0.001	<0.001		
Born at home	coef.	-0.170***	-0.157***	-0.185***	8.1%***	15.5%***
(vs institutional birth)	p-val	<0.001	<0.001	<0.001		
Birth interval <24m	coef.	-0.189***	-0.146***	-0.205***	8.1%***	39.6%***
(vs >24m)	p-val	<0.001	<0.001	<0.001		
3–4 Children born	coef.	-0.042***	-0.022***	-0.054***	26.2%***	152.4%***
(vs 1–2 children)	p-val	<0.001	(0.008)	<0.001		
5+ Children born	coef.	-0.112***	-0.112***	-0.103***	-9.8%*	-12.2%
(vs 1–2 children)	p-val	<0.001	<0.001	<0.001		
Teenage mother (<20 yrs)	coef.	-0.145***	-0.126***	-0.154***	8.2%**	28.2%**
(vs >19 years)	p-val	<0.001	<0.001	<0.001		
Mother <145 cm	coef.	-0.948***	-0.957***	-0.942***	-0.8%	-1.9%
(vs >155 cm)	p-val	<0.001	<0.001	<0.001		
Mother 145–150 cm	coef.	-0.628***	-0.625***	-0.630***	-0.2%	-0.6%
(vs >155 cm)	p-val	<0.001	<0.001	<0.001		
Mother 150-155cm	coef.	-0.368***	-0.375***	-0.363***	-1.6%	-3.6%
(vs >155cm)	p-val	<0.001	<0.001	<0.001		
Improved latrine	coef.	0.195***	0.169***	0.209***	8.9%**	29.4%***
(vs no latrine)	p-val	<0.001	<0.001	<0.001		
Unimproved latrine	coef.	0.032***	0.036***	0.029***	5.1%	13.9%
(vs no latrine)	p-val	<0.001	<0.001	<0.001		
Improved water	coef.	0.015**	-0.002	0.028***	93.3%***	680.0%***
(vs unimproved water)	p-val	0.015	0.790	<0.001		
Rural	coef.	-0.088***	-0.064***	-0.104***	18.2%***	62.5%***
(vs urban)	p-val	<0.001	<0.001	<0.001		
Boy	coef.	-0.135***	-0.231***	-0.067***	-50.4%***	-71.0%***
(vs girl)	p-val	<0.001	<0.001	<0.001		
Father missing	coef.	0.008	-0.010	0.029***	-49.3%***	-69.9%**
(vs present)	p-val	0.357	0.440	0.006		
R-squared		0.187	0.160	0.174		

Notes:

*** p<0.01,

** p<0.05,

* p<0.1 p-values are based on standard errors adjusted for clustering at the DHS cluster level. Regressions include dummy variables for each of the 125 surveys (country-year fixed effects) and dummies each month of children’s age.

^a^. These are the percentage differences between the coefficients reported in columns (1) and (3) and (2) and (3), with Wald tests of the null hypothesis of coefficient equality across the regression equations.

**Fig 3:**
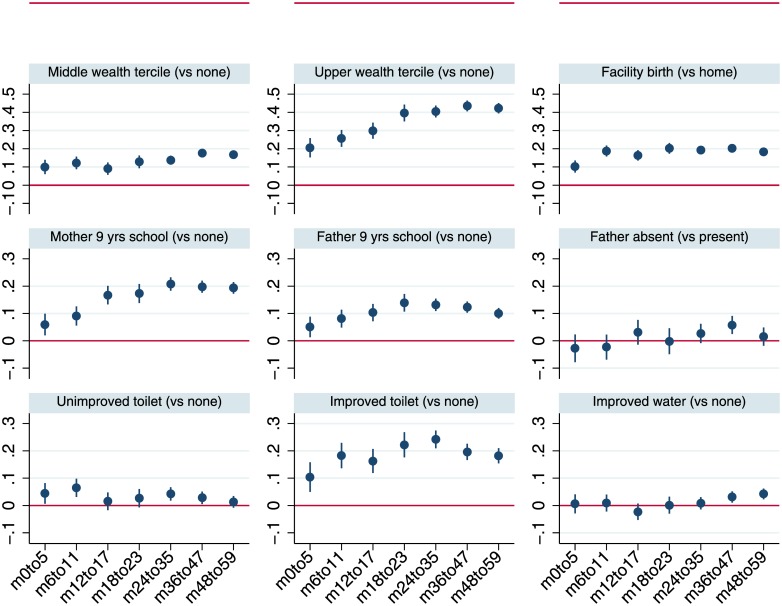
Least squares estimates for HAZ scores by age-restricted sub-samples: Household wealth, health facility access, parental education, household sanitation and water variables. Notes: The figure reports coefficients and 95% confidence intervals based on cluster-adjusted standard errors from multivariable least squares regressions of HAZ against all the variables listed in Table 1, as well as country-year fixed effects and dummy variables for every month of child age. Samples sizes for the regressions estimated for the various age groups are 67,384 (0–5 months; *m0to5*), 75,965 (6–11 months; *m6to11*), 76,711 (12–17 months; *m12to17*), 68,694 (18–23 months; *m18to23*), 129,174 (24–35 months; *m24-35*), 138,342 (36–47 months; *m36to47*), 132,242 (48–59 months; *m48to59*).

**Fig 4:**
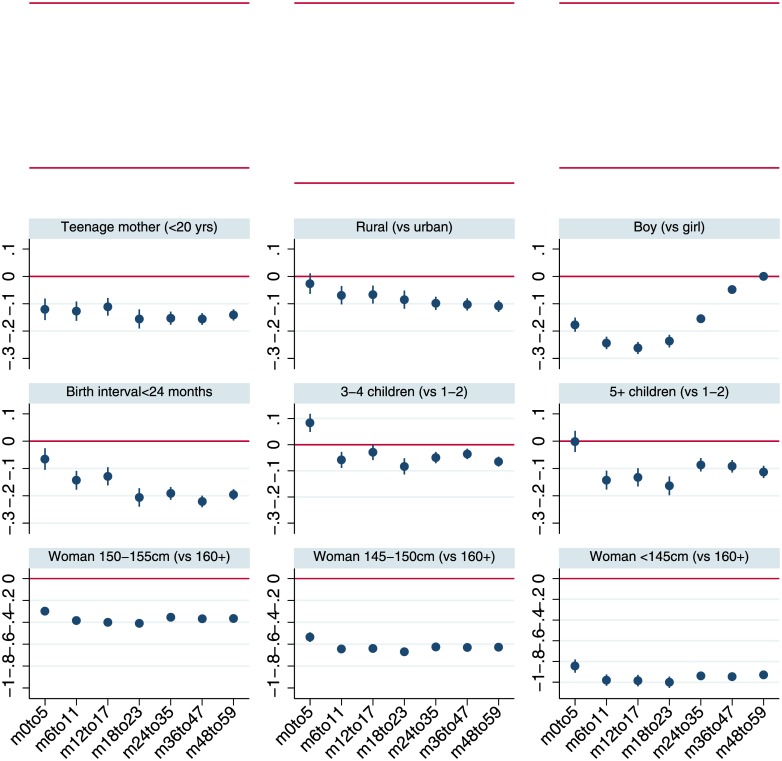
Least squares estimates for HAZ scores by age-restricted sub-samples: Child, maternal and household demographic indicators and maternal height. Notes: The figure reports coefficients and 95% confidence intervals based on cluster-adjusted standard errors from multivariable least squares regressions of HAZ against all the variables listed in Table 1, as well as country-year fixed effects and dummy variables for every month of child age. Samples sizes for the regressions estimated for the various age groups are 67,384 (0–5 months; *m0to5*), 75,965 (6–11 months; *m6to11*), 76,711 (12–17 months; *m12to17*), 68,694 (18–23 months; *m18to23*), 129,174 (24–35 months; *m24-35*), 138,342 (36–47 months; *m36to47*), 132,242 (48–59 months; *m48to59*).

Fig 3 plots the coefficients from multivariable regressions for plausible protective factors by more disaggregated sub-samples of child age with 95% confidence intervals, while Fig 4 does the same for adverse risk factors. Although the analysis is not longitudinal, the figures provide a perspective on the time path of the associations of stunting and its correlates. Fig 3 suggests that the associations of wealth, particularly for households in the upper tercile, increase steadily as children age. Broadly similar tendencies for coefficients to increase with age also hold for maternal education and improved sanitation. The coefficients plotted in Fig 4 reiterate the pattern observed in Fig 1, that in the first 1000 days boys have greater growth retardation but at slightly older ages there is no difference in HAZ compared to girls.

**Fig 5:**
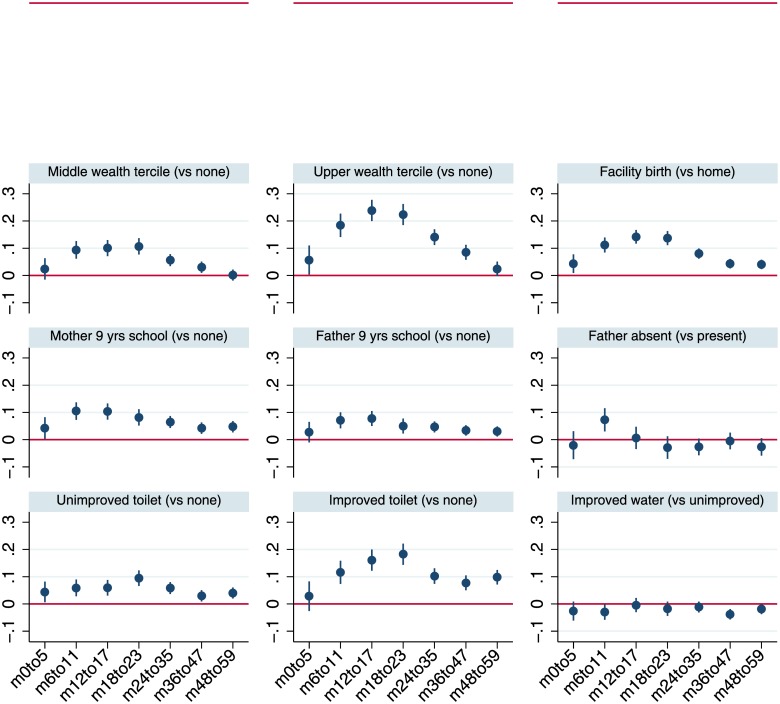
Least squares estimates for WHZ scores by age-restricted sub-samples: Household wealth, health facility access, parental education, household sanitation and water variables. Notes: The figure reports coefficients and 95% confidence intervals based on cluster-adjusted standard errors from multivariable least squares regressions of HAZ against all the variables listed in Table 1, as well as country-year fixed effects and dummy variables for every month of child age. Samples sizes for the regressions estimated for the various age groups are 67,384 (0–5 months; *m0to5*), 75,965 (6–11 months; *m6to11*), 76,711 (12–17 months; *m12to17*), 68,694 (18–23 months; *m18to23*), 129,174 (24–35 months; *m24-35*), 138,342 (36–47 months; *m36to47*), 132,242 (48–59 months; *m48to59*).

**Fig 6:**
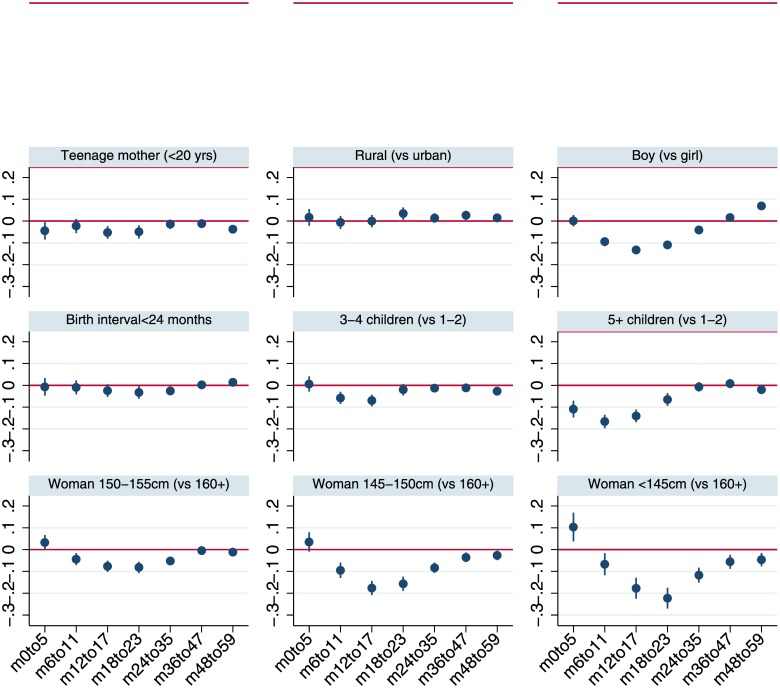
Least squares estimates for WHZ scores by age-restricted sub-samples: Child, maternal and household demographic indicators and maternal height. Notes: The figure reports coefficients and 95% confidence intervals based on cluster-adjusted standard errors from multivariable least squares regressions of HAZ against all the variables listed in Table 1, as well as country-year fixed effects and dummy variables for every month of child age. Samples sizes for the regressions estimated for the various age groups are 67,384 (0–5 months; *m0to5*), 75,965 (6–11 months; *m6to11*), 76,711 (12–17 months; *m12to17*), 68,694 (18–23 months; *m18to23*), 129,174 (24–35 months; *m24-35*), 138,342 (36–47 months; *m36to47*), 132,242 (48–59 months; *m48to59*).

Table 3 and Figs 5 and 6 replicate the above analysis for WHZ scores. Given that WHZ is not a cumulative measure, and that WHZ reaches a nadir at approximately 12 months of age in this DHS sample, one would expect associations with various underlying determinants to be larger for younger children. That is almost universally what we observe, and the differences are generally statistically significant. The coefficients on wealth terciles and parental education, for example, are 44 to 65% larger in the 0–23 month sub-sample compared to the 24–59 month sub-sample. Using the commonly reported 0–59 month sub-sample also leads to substantial attenuation relative to the 0–23 month sub-sample for these coefficients, with the partial exposure bias varying between 24 and 44%. Large differences are observed for the coefficients of many other variables, including being born at home, having larger numbers of children, maternal height dummies, sanitation indicators and the boy dummy. Figs 5 and 6 reveal patterns that are highly consistent with the WHZ-child age graph depicted in Fig 2: coefficients increase in magnitude from 0–5 months to 12–17 months where they typically peak, before steadily declining for older age brackets.

**Table 3:** Least squares regressions of child WHZ against standard explanatory variables for children aged 0–59 months, 0–23 months and 24–59 months from 57 developing countries.

		(1)	(2)	(3)	Differences across samples:^a^	
		N = 699,421	N = 288,754	N = 410,667	(3) minus (1)	(3) minus (2)
		0–59 months	0–23 months	24–59 months		
Variable						
(base category)						
Middle wealth tercile	coef.	0.052***	0.082***	0.029***	-44.0%***	-65.0%***
(vs lowest)	p-val	<0.001	<0.001	<0.001		
Upper wealth tercile	coef.	0.121***	0.179***	0.084***	-30.2%***	-52.5%***
(vs lowest)	p-val	<0.001	<0.001	<0.001		
Mother 9 years school	coef.	0.070***	0.085***	0.054***	-26.1%***	-40.0%***
(vs none)	p-val	<0.001	<0.001	<0.001		
Father 9 years school	coef.	0.046***	0.059***	0.036***	-24.4%**	-44.2%**
(vs none)	p-val	<0.001	<0.001	<0.001		
Born at home	coef.	-0.077***	-0.107***	-0.054***	-27.8%***	-47.7%***
(vs institutional birth)	p-val	<0.001	<0.001	<0.001		
Birth interval <24m	coef.	-0.002	-0.020**	-0.003	NA	NA
(vs >24m)	p-val	(0.698)	(0.017)	(0.521)		
3–4 Children born	coef.	-0.031***	-0.038***	-0.018***	-48.3%	-59.4%**
(vs 1–2 children)	p-val	<0.001	<0.001	(0.001)		
5+ Children born	coef.	-0.051***	-0.122***	-0.004	-94.1%***	-97.5%***
(vs 1–2 children)	p-val	<0.001	<0.001	(0.518)		
Teenage mother (<20 yrs)	coef.	-0.034***	-0.045***	-0.021***	-42.4%**	-59.5%**
(vs >19 years)	p-val	<0.001	<0.001	(0.001)		
Mother <145 cm	coef.	-0.080***	-0.091***	-0.072***	-13.2%	-27.2%
(vs >155 cm)	p-val	<0.001	<0.001	<0.001		
Mother 145–150 cm	coef.	-0.070***	-0.099***	-0.049***	-30.0%**	-51.0%***
(vs >155 cm)	p-val	<0.001	<0.001	<0.001		
Mother 150-155cm	coef.	-0.032***	-0.043***	-0.024***	-29.4%***	-48.9%**
(vs >155cm)	p-val	<0.001	<0.001	<0.001		
Improved latrine	coef.	0.104***	0.127***	0.091***	-14.7%**	-31.5%***
(vs no latrine)	p-val	<0.001	<0.001	<0.001		
Unimproved latrine	coef.	0.051***	0.064***	0.042***	-14.0%**	-29.5%**
(vs no latrine)	p-val	<0.001	<0.001	<0.001		
Improved water	coef.	-0.022***	-0.021***	-0.024***	0.0%	5.2%
(vs unimproved water)	p-val	<0.001	(0.010)	<0.001		
Rural	coef.	0.014**	0.010	0.017**	25.0%	66.7%
(vs urban)	p-val	(0.028)	(0.262)	(0.019)		
Boy	coef.	-0.026***	-0.085***	0.016***	-176.0%***	-121.6%***
(vs girl)	p-val	<0.001	<0.001	<0.001		
Father missing	coef.	-0.000	0.014	-0.019**	1300.0%***	-240.0%**
(vs present)	p-val	(0.983)	(0.218)	(0.037)		
R-squared		0.120	0.122	0.126		

Notes:

*** p<0.01,

** p<0.05,

* p<0.1 p-values are based on standard errors adjusted for clustering at the DHS cluster level. Regressions include dummy variables for each of the 125 surveys (country-year fixed effects) and dummies each month of children’s age.

^a^. These are the percentage differences between the coefficients reported in columns (1) and (3) and (2) and (3), with Wald tests of the null hypothesis of coefficient equality across the regression equations.

Results reported in S2 and S3 Tables quantify analogous patterns for moderate stunting (HAZ<-2) and wasting (WHZ<-2) using linear probability models, since these dichotomous indicators are widely reported in the literature. In S2 Table for example, switching from the conventional 0–59 month sample to the 24–59 month sample increases the absolute value of all the wealth and maternal education coefficients in HAZ regressions by approximately 25%.

We also replicated Tables 2 and 3 for each major region. The region-specific results for HAZ and WHZ show very similar patterns to those reported in Tables 2 and 3. For brevity, S1 and S2 Figs summarize coefficient differences for three key variables (upper wealth tercile, maternal education and improved latrine) from HAZ and WHZ regressions for the 0–23 and 24–59 month samples. The region-specific results for HAZ and WHZ show very similar patterns to those reported in Tables 2 and 3: larger coefficients in the older sample of children (24–59 month) for variables such as household wealth, parental education and sanitation holds in all major regions when HAZ is the dependent variable, and an opposite pattern when WHZ is the dependent variable. Our main results are therefore not being driven by any particular region, and are likely to hold across a wide range of national and regional sub-samples.

S4 Table also addresses concerns about issue with HAZ stemming from the fact that growth reference standard deviations increase with age. Following the suggestion of [4], S4 Table uses the absolute height deficit in centimeters from the WHO 2006 growth standard’s median heights (HAD). The percentage differences between the 0–23 and 24–59 month samples are even larger than the analogous differences for HAZ reported in Table 2, suggesting our key messages are not restricted to measurements based on HAZ.

## Conclusions

Despite the broader programmatic influence of seminal research on the importance of growth faltering and wasting in the first 1000 days of life [1, 2], experimental and observational research on linear growth and wasting often only measures partial exposure to underlying determinants of interest without paying due attention to the different age dynamics of growth faltering as defined by HAZ and WAZ or by HAD. The results in this study show that the multivariable regression associations between these indicators and various underlying determinants of nutrition using DHS data are very sensitive to age restrictions, with patterns entirely consistent with the partial exposure bias hypothesis.

The clearest limitation of these results is that they are based on cross sectional observational data, implying that these coefficients could suffer from the omission of relevant confounding factors and various types of measurement error, and that any inferences on the dynamics of growth faltering and wasting must be inferred from comparisons across cohorts rather than within cohorts. However, the primary objective of our analysis was to gauge the sensitivity of findings inferred from an already extensive observational literature on the underlying determinants of nutrition. This literature exists because in contrast to the evidence base on specific nutrition programs, experimental evaluations of the nutritional impacts of many underlying factors are costly and difficult to implement. For example, assessing the nutritional impacts of parental schooling would require an extremely extensive and prolonged multi-generational experimental design [8]. Hence there are very few experimental studies assessing the nutritional impacts of programs targeting parental education, household income/assets, access to health services or family planning. In the absence of many such experiments, researchers and policymakers heavily rely on observational analyses to investigate these factors or to infer their role in modelling scenarios; this paper set out to assess how sensitive such analyses are to partial exposure bias.

These findings have important implications for future research. For observational analyses of linear growth indicators, minimizing partial exposure bias in studies of underlying determinants *generally* requires focusing regression analysis on children aged 24 months or older who have completed the first 1000 days of heightened vulnerability to various nutritional insults. One necessary exception would be analyses of the associations between HAZ/stunting and children’s diets [34, 35], since the DHS now only measures dietary outcomes for children 0–23 months (previously many surveys had recorded this information for children 0–35 months). Yet even in this case researchers should test sensitivity to the use of older sub-samples (e.g. 18–23 months) and arguably use sub-samples of older children as the preferred result, as in [36]. Another exception would be analyses focused on specific stages of a child’s growth process, such as studies exploring determinants of postnatal HAZ and its associations with maternal nutrition or prenatal care.

For analyses of WHZ or wasting, the opposite strategy is recommended: regression analyses should generally focus on children 0–23 months of age, since these children are much more vulnerable to the various recent insults that influence weight-based nutrition outcomes. At the very least, studies should stratify their analyses by appropriate age brackets, and acknowledge that partial exposure bias can be an important influence on estimated coefficients.

These recommendations can easily be implemented with the data often collected in standard surveys. More indirectly, these results also have implications for experimental research and thus for the samples chosen or for the necessary duration of the studies. Nutrition-specific interventions often do give very close consideration to the age dynamics of stunting, but not always. For example, many analyses of nutrition-sensitive interventions, such as sanitation trials, often pay limited attention to this issue, especially when nutrition indicators constitute secondary outcomes of interest [27].

These results have important implications for the policy messages inferred from existing research on the determinants of child nutrition, which mostly uses samples of children aged 0–59 months. Our findings suggest that many previous studies have underestimated the impacts of a wide range of underlying determinants on linear growth, particularly parental education and wealth, income and economic growth [4–7]. Interestingly, our analysis of the determinants of child weight for height also suggests that wealth and parental education have much stronger associations with WHZ and wasting in the more appropriate 0–23 month old sample.

In summary, we argue that the analytical implications of children’s heightened vulnerability to nutritional insults in the first 1000 days of life have not been appropriately integrated into the large and influential literature engaged in observational analyses of the underlying determinants of child nutrition outcomes. Doing so yields important new results, with household wealth, parental education and many other underlying determinants having stronger associations with stunting and wasting than previous analyses would suggest.

## Supporting information

S1 Table Stunting (HAZ<-2) and wasting (WHZ<-2) rates for children 0–59 months, 0–23 months and 24–59 months for 125 Demographic Health Surveys. (DOCX)

S2 Table Linear probability regressions of child stunting against standard explanatory variables for children aged 0–59 months, 0–23 months and 24–59 months from 57 developing countries. (DOCX)

S3 Table Linear probability regressions of child wasting (WHZ<-2) against standard explanatory variables for children aged 0–59 months, 0–23 months and 24–59 months from 57 developing countries. (DOCX)

S4 Table Least squares regressions of child height for age deficits (cm) against standard explanatory variables for children aged 0–59 months, 0–23 months and 24–59 months from 57 developing countries. (DOCX)

S1 Fig Examples of coefficient differences from HAZ regressions across the 0–23 and 24–59 month samples for the four largest regions in the full DHS sample. Note: SSA (Sub-Saharan Africa); SAS (South Asia), LAC (Latin America and the Caribbean and MNA (Middle East and North Africa)
(TIF)

S2 Fig Examples of coefficient differences from WHZ regressions across the 0–23 and 24–59 month samples for the four largest regions in the full DHS sample. Note: SSA (Sub-Saharan Africa); SAS (South Asia), LAC (Latin America and the Caribbean and MNA (Middle East and North Africa)
(TIF)
