# Supplementary material for: The timing of growth faltering has important implications for observational analyses of the underlying determinants of nutrition outcomes
Source: PLoS One. 2018 Apr 25;13(4):e0195904. doi: 10.1371/journal.pone.0195904 (PMC5919068; doi:10.1371/journal.pone.0195904)
Supplement: S1 Table — (DOCX) [file pone.0195904.s001.docx]

**S1 Table. Stunting (HAZ<-2) and wasting (WHZ<-2) rates for children 0-59 months, 0-23 months and 24-59 months for 125 Demographic Health Surveys**

|  |  |  | Stunting rates (HAZ<-2) | | | Wasting rates (WHZ<-2) | | |
| --- | --- | --- | --- | --- | --- | --- | --- | --- |
| Country | Year | N | 0-59m | 0-23 m | 24-59 m | 0-59 m | 0-23 m | 24-59 m |
| Albania | 2009 | 1,349 | 20.9% | 27.9% | 17.6% | 9.1% | 11.1% | 8.1% |
| Armenia | 2000 | 1,510 | 17.0% | 11.9% | 19.9% | 2.4% | 4.4% | 1.2% |
| Armenia | 2005 | 1,212 | 18.3% | 19.6% | 17.4% | 5.2% | 6.3% | 4.4% |
| Azerbaijan | 2006 | 1,932 | 26.2% | 16.3% | 33.5% | 7.1% | 11.5% | 3.9% |
| Bangladesh | 2007 | 5,051 | 43.2% | 31.4% | 50.6% | 17.3% | 20.3% | 15.4% |
| Bangladesh | 2011 | 7,343 | 41.2% | 34.4% | 45.2% | 15.5% | 15.0% | 15.8% |
| Benin | 2001 | 4,349 | 38.2% | 26.0% | 47.6% | 9.2% | 16.4% | 3.7% |
| Benin | 2012 | 9,344 | 46.8% | 38.3% | 52.1% | 16.5% | 18.6% | 15.3% |
| Bolivia | 2003 | 8,876 | 32.3% | 25.2% | 36.4% | 1.6% | 2.7% | 1.0% |
| Bolivia | 2008 | 7,384 | 27.4% | 20.7% | 32.0% | 1.4% | 2.6% | 0.5% |
| Burkina Faso | 1993 | 4,325 | 40.6% | 26.8% | 51.4% | 15.3% | 25.2% | 7.7% |
| Burkina Faso | 1999 | 4,490 | 45.2% | 29.9% | 56.8% | 15.3% | 25.3% | 7.9% |
| Burkina Faso | 2003 | 8,193 | 43.2% | 31.2% | 51.9% | 21.5% | 32.7% | 13.5% |
| Burkina Faso | 2010 | 6,526 | 34.6% | 25.1% | 41.2% | 15.9% | 25.3% | 9.3% |
| Burundi | 2010 | 3,339 | 58.1% | 47.6% | 65.8% | 5.9% | 9.1% | 3.5% |
| Cambodia | 2000 | 3,329 | 49.4% | 36.1% | 57.7% | 16.9% | 20.0% | 15.0% |
| Cambodia | 2005 | 3,582 | 42.7% | 30.4% | 51.0% | 8.6% | 11.4% | 6.7% |
| Cameroon | 2004 | 3,067 | 35.9% | 29.2% | 41.2% | 6.2% | 9.2% | 3.8% |
| Cameroon | 2011 | 4,901 | 32.0% | 23.8% | 38.7% | 6.0% | 9.7% | 3.0% |
| Chad | 2004 | 4,279 | 45.3% | 29.2% | 56.5% | 16.2% | 24.1% | 10.8% |
| Colombia | 1995 | 4,312 | 19.5% | 15.3% | 22.3% | 1.7% | 2.0% | 1.5% |
| Colombia | 2010 | 14,740 | 12.4% | 12.1% | 12.6% | 0.9% | 1.3% | 0.7% |
| Comoros | 2012 | 2,315 | 30.3% | 29.7% | 30.7% | 11.1% | 14.3% | 8.8% |
| Congo, Rep. | 2005 | 3,624 | 30.1% | 23.7% | 35.1% | 7.8% | 10.1% | 6.0% |
| Congo, Rep. | 2012 | 4,175 | 22.7% | 19.9% | 25.0% | 5.6% | 7.4% | 4.2% |
| Congo (DRC) | 2007 | 3,189 | 44.5% | 29.2% | 54.4% | 10.0% | 14.3% | 7.2% |
| Congo (DRC) | 2014 | 7,904 | 43.0% | 29.4% | 52.7% | 7.9% | 10.3% | 6.2% |
| Cote d'Ivoire | 2011 | 3,058 | 29.7% | 22.9% | 35.2% | 7.7% | 12.1% | 4.1% |
| Dominican Republic | 2013 | 3,076 | 6.9% | 8.1% | 6.0% | 2.1% | 2.5% | 1.9% |
| Egypt | 2000 | 10,377 | 24.0% | 24.6% | 23.7% | 3.0% | 4.9% | 1.7% |
| Egypt | 2003 | 6,018 | 20.0% | 25.0% | 16.7% | 5.2% | 6.4% | 4.4% |
| Egypt | 2005 | 12,503 | 23.6% | 26.3% | 21.8% | 4.9% | 7.3% | 3.4% |
| Egypt | 2008 | 9,700 | 30.0% | 27.4% | 31.9% | 7.5% | 8.2% | 7.0% |
| Egypt | 2014 | 13,860 | 22.1% | 21.4% | 22.6% | 8.8% | 10.4% | 7.6% |
| Ethiopia | 2005 | 3,838 | 50.6% | 38.6% | 58.0% | 12.3% | 16.8% | 9.5% |
| Ethiopia | 2011 | 9,482 | 44.3% | 29.3% | 53.9% | 9.8% | 14.5% | 6.8% |
| Gabon | 2000 | 3,296 | 26.0% | 22.9% | 28.5% | 4.1% | 6.0% | 2.7% |
| Gabon | 2012 | 3,171 | 16.8% | 15.1% | 18.1% | 3.7% | 4.0% | 3.5% |
| Ghana | 1998 | 2,740 | 31.4% | 20.0% | 40.2% | 9.7% | 15.6% | 5.1% |
| Ghana | 2003 | 3,025 | 35.1% | 25.3% | 42.1% | 8.4% | 14.0% | 4.4% |
| Ghana | 2008 | 2,360 | 27.6% | 19.8% | 33.1% | 9.0% | 16.0% | 4.1% |
| Guatemala | 1995 | 8,269 | 55.7% | 44.0% | 64.2% | 3.8% | 5.1% | 2.8% |
| Guatemala | 1999 | 3,758 | 53.9% | 44.5% | 60.0% | 2.9% | 5.1% | 1.5% |
| Guinea | 1999 | 4,123 | 33.8% | 22.9% | 41.2% | 9.6% | 16.5% | 5.0% |
| Guinea | 2005 | 2,566 | 39.0% | 24.8% | 50.2% | 11.2% | 16.4% | 7.1% |
| Guinea | 2012 | 3,032 | 31.1% | 19.6% | 39.7% | 10.5% | 16.1% | 6.2% |
| Guyana | 2009 | 1,534 | 18.3% | 19.5% | 17.6% | 5.4% | 7.1% | 4.4% |
| Haiti | 2000 | 5,444 | 27.8% | 21.3% | 32.5% | 5.6% | 8.6% | 3.5% |
| Haiti | 2006 | 2,506 | 29.1% | 24.8% | 32.3% | 10.4% | 12.9% | 8.6% |
| Haiti | 2012 | 3,894 | 21.0% | 14.9% | 25.9% | 5.0% | 7.1% | 3.3% |
| Honduras | 2006 | 8,396 | 29.6% | 22.1% | 33.8% | 1.2% | 2.0% | 0.7% |
| Honduras | 2012 | 9,426 | 22.5% | 16.0% | 27.2% | 1.4% | 2.0% | 0.9% |
| India | 2006 | 40,990 | 47.7% | 38.8% | 53.2% | 20.0% | 26.4% | 16.1% |
| Jordan | 1997 | 5,558 | 11.0% | 10.8% | 11.1% | 2.2% | 2.9% | 1.8% |
| Kazakhstan | 1995 | 732 | 20.6% | 18.5% | 25.1% | 7.2% | 9.5% | 2.3% |
| Kazakhstan | 1999 | 564 | 12.9% | 11.3% | 13.9% | 2.5% | 3.9% | 1.7% |
| Kenya | 1998 | 4,435 | 38.2% | 30.9% | 43.8% | 7.2% | 9.8% | 5.2% |
| Kenya | 2003 | 4,339 | 36.2% | 31.1% | 40.2% | 5.8% | 8.1% | 4.1% |
| Kenya | 2009 | 5,015 | 35.2% | 32.1% | 37.3% | 6.6% | 7.7% | 5.9% |
| Kyrgyz Republic | 1999 | 978 | 32.4% | 27.4% | 42.8% | 3.3% | 4.3% | 1.1% |
| Kyrgyz Republic | 2012 | 4,005 | 18.0% | 14.5% | 20.7% | 2.8% | 3.8% | 1.9% |
| Lesotho | 2004 | 1,344 | 42.9% | 35.5% | 48.8% | 5.4% | 8.1% | 3.2% |
| Lesotho | 2009 | 1,569 | 38.3% | 28.6% | 46.5% | 4.1% | 6.0% | 2.4% |
| Liberia | 2007 | 4,150 | 38.0% | 25.3% | 47.2% | 7.5% | 12.5% | 3.9% |
| Liberia | 2013 | 3,099 | 30.8% | 22.2% | 37.5% | 6.0% | 9.6% | 3.1% |
| Madagascar | 1997 | 2,886 | 55.7% | 49.4% | 71.0% | 10.0% | 11.4% | 6.8% |
| Madagascar | 2004 | 4,431 | 52.6% | 45.9% | 57.7% | 15.3% | 19.2% | 12.3% |
| Malawi | 2004 | 8,010 | 51.9% | 44.7% | 57.8% | 6.3% | 8.4% | 4.4% |
| Malawi | 2010 | 4,513 | 47.3% | 40.3% | 52.3% | 4.1% | 6.6% | 2.4% |
| Mali | 2006 | 10,699 | 38.0% | 29.3% | 44.7% | 15.6% | 23.4% | 9.6% |
| Mali | 2013 | 4,280 | 38.4% | 27.4% | 45.0% | 12.7% | 18.4% | 9.2% |
| Moldova | 2005 | 1,310 | 10.7% | 10.9% | 10.6% | 5.1% | 5.2% | 5.1% |
| Morocco | 1992 | 4,495 | 30.2% | 23.4% | 34.6% | 2.6% | 3.4% | 2.1% |
| Morocco | 2004 | 5,440 | 23.0% | 22.3% | 23.4% | 10.3% | 9.2% | 10.9% |
| Mozambique | 1997 | 3,264 | 45.4% | 36.4% | 67.2% | 12.0% | 14.2% | 6.8% |
| Mozambique | 2003 | 7,621 | 46.8% | 37.7% | 54.0% | 5.3% | 7.8% | 3.4% |
| Mozambique | 2011 | 9,141 | 42.8% | 37.9% | 46.6% | 6.1% | 9.5% | 3.6% |
| Namibia | 2002 | 2,522 | 34.6% | 33.0% | 36.2% | 9.4% | 9.7% | 9.1% |
| Namibia | 2007 | 3,553 | 28.5% | 24.6% | 32.1% | 7.6% | 9.2% | 6.1% |
| Namibia | 2013 | 1,684 | 21.2% | 16.2% | 25.9% | 8.3% | 11.5% | 5.2% |
| Nepal | 2006 | 5,181 | 49.2% | 32.4% | 59.3% | 12.7% | 17.1% | 10.1% |
| Nepal | 2011 | 2,322 | 39.9% | 25.2% | 49.3% | 11.1% | 16.7% | 7.5% |
| Nicaragua | 1998 | 6,428 | 30.5% | 23.2% | 34.8% | 3.0% | 4.8% | 1.9% |
| Nicaragua | 2011 | 5,633 | 24.8% | 18.6% | 28.7% | 2.1% | 3.6% | 1.2% |
| Niger | 2006 | 3,644 | 54.8% | 41.0% | 65.1% | 12.8% | 18.6% | 8.5% |
| Niger | 2012 | 4,706 | 43.3% | 31.5% | 51.3% | 18.0% | 24.6% | 13.5% |
| Nigeria | 2003 | 4,318 | 42.8% | 33.9% | 49.8% | 11.3% | 16.9% | 6.9% |
| Nigeria | 2008 | 18,847 | 40.6% | 36.0% | 43.9% | 14.3% | 17.5% | 12.0% |
| Nigeria | 2013 | 24,346 | 36.5% | 28.9% | 41.9% | 18.2% | 23.9% | 14.2% |
| Pakistan | 2013 | 3,082 | 44.7% | 37.6% | 49.1% | 10.5% | 16.9% | 6.7% |
| Peru | 1992 | 7,404 | 37.5% | 26.2% | 44.9% | 1.8% | 2.6% | 1.3% |
| Peru | 1996 | 13,463 | 31.1% | 24.5% | 35.4% | 1.5% | 2.3% | 1.0% |
| Peru | 2000 | 11,076 | 31.4% | 23.5% | 36.1% | 1.1% | 1.5% | 0.8% |
| Peru | 2008 | 9,277 | 27.8% | 24.3% | 29.9% | 0.8% | 1.0% | 0.8% |
| Peru | 2009 | 8,458 | 23.6% | 21.3% | 25.1% | 0.6% | 0.7% | 0.5% |
| Peru | 2010 | 8,039 | 22.7% | 21.7% | 23.4% | 0.7% | 1.0% | 0.5% |
| Peru | 2011 | 8,073 | 19.3% | 19.4% | 19.3% | 0.4% | 0.6% | 0.3% |
| Peru | 2012 | 8,092 | 18.5% | 18.5% | 18.5% | 0.7% | 1.0% | 0.5% |
| Rwanda | 2000 | 5,981 | 48.0% | 35.8% | 57.7% | 8.4% | 12.3% | 5.3% |
| Rwanda | 2005 | 3,612 | 50.9% | 39.1% | 59.6% | 4.7% | 7.7% | 2.5% |
| Rwanda | 2010 | 3,995 | 44.1% | 34.6% | 49.9% | 2.9% | 4.9% | 1.6% |
| Sao Tome & Principe | 2008 | 1,457 | 28.9% | 37.4% | 23.6% | 11.0% | 10.4% | 11.3% |
| Senegal | 1993 | 4,387 | 33.3% | 23.1% | 40.4% | 9.2% | 12.7% | 6.7% |
| Senegal | 2005 | 2,698 | 19.4% | 14.8% | 23.2% | 8.3% | 10.5% | 6.6% |
| Senegal | 2011 | 3,606 | 28.0% | 23.2% | 31.4% | 9.9% | 11.2% | 8.9% |
| Sierra Leone | 2008 | 1,971 | 36.6% | 26.3% | 44.7% | 10.2% | 11.9% | 8.9% |
| Sierra Leone | 2013 | 4,028 | 37.8% | 31.3% | 42.2% | 9.4% | 13.3% | 6.7% |
| Swaziland | 2007 | 1,968 | 27.6% | 26.5% | 28.4% | 2.5% | 4.0% | 1.4% |
| Tajikistan | 2012 | 4,530 | 26.6% | 23.3% | 28.9% | 9.9% | 14.6% | 6.6% |
| Tanzania | 1992 | 6,293 | 49.8% | 40.3% | 57.9% | 7.5% | 9.7% | 5.7% |
| Tanzania | 1996 | 5,204 | 49.6% | 40.0% | 57.6% | 8.3% | 11.8% | 5.4% |
| Tanzania | 2005 | 7,075 | 44.0% | 35.5% | 50.9% | 3.5% | 5.1% | 2.2% |
| Tanzania | 2010 | 6,709 | 41.7% | 35.4% | 46.6% | 4.9% | 7.7% | 2.8% |
| Timor-Leste | 2010 | 7,019 | 57.7% | 48.6% | 62.9% | 18.6% | 19.2% | 18.3% |
| Uganda | 2001 | 4,974 | 44.5% | 37.2% | 50.5% | 5.0% | 8.4% | 2.2% |
| Uganda | 2006 | 2,344 | 37.6% | 30.1% | 43.4% | 6.5% | 11.9% | 2.4% |
| Uganda | 2011 | 2,044 | 33.4% | 27.6% | 38.2% | 4.9% | 8.3% | 2.2% |
| Zambia | 1992 | 4,820 | 46.5% | 36.0% | 55.4% | 6.3% | 8.7% | 4.3% |
| Zambia | 1996 | 5,367 | 48.8% | 37.0% | 58.3% | 5.1% | 8.9% | 2.1% |
| Zambia | 2002 | 5,320 | 52.8% | 42.7% | 60.7% | 6.2% | 10.1% | 3.2% |
| Zambia | 2007 | 4,893 | 45.6% | 39.0% | 50.6% | 5.4% | 7.7% | 3.7% |
| Zambia | 2014 | 11,219 | 39.8% | 36.0% | 42.3% | 6.1% | 7.8% | 5.0% |
| Zimbabwe | 1999 | 2,596 | 32.4% | 27.8% | 35.9% | 7.7% | 10.8% | 5.4% |
| Zimbabwe | 2006 | 3,875 | 34.5% | 31.0% | 37.0% | 6.9% | 8.7% | 5.7% |
| Zimbabwe | 2011 | 4,235 | 31.2% | 23.5% | 38.2% | 3.2% | 5.0% | 1.6% |
| Total Sample |  | 699,421 | 36.1% | 29.4% | 40.8% | 8.4% | 11.5% | 6.3% |
