# Supplementary material for: The timing of growth faltering has important implications for observational analyses of the underlying determinants of nutrition outcomes
Source: PLoS One. 2018 Apr 25;13(4):e0195904. doi: 10.1371/journal.pone.0195904 (PMC5919068; doi:10.1371/journal.pone.0195904)
Supplement: S3 Table — (DOCX) [file pone.0195904.s003.docx]

**S3 Table. Linear probability regressions of child wasting (WHZ<-2) against standard explanatory variables for children aged 0-59 months, 0-23 months and 24-59 months from 57 developing countries**

|  |  | (1) | (2) | (3) | Differences across samples: ^a^ | |
| --- | --- | --- | --- | --- | --- | --- |
|  |  | N=699,421 | N=288,754 | N=410,667 | (3) minus (1) | (3) minus (2) |
|  |  | 0-59 months | 0-23 months | 24-59 months |  |  |
| Variable |  |  |  |  |  |  |
| (base category) |  |  |  |  |  |  |
|  |  |  |  |  |  |  |
| Middle wealth tercile | coef. | -0.010*** | -0.012*** | -0.009*** | -11.11% | -27.27% |
| (vs none) | p-val | <0.001 | <0.001 | <0.001 |  |  |
| Upper wealth tercile | coef. | -0.016*** | -0.023*** | -0.012*** | -26.67% | -47.62% |
| (vs none) | p-val | <0.001 | <0.001 | <0.001 |  |  |
| Mother 9 years school | coef. | -0.008*** | -0.011*** | -0.005*** | -25.00% | -45.45% |
| (vs none) | p-val | <0.001 | <0.001 | <0.001 |  |  |
| Father 9 years school | coef. | -0.008*** | -0.008*** | -0.008*** | 0.00% | -11.11% |
| (vs none) | p-val | <0.001 | <0.001 | <0.001 |  |  |
| Born at home | coef. | 0.011*** | 0.014*** | 0.008*** | -20.00% | -38.46% |
| (vs institutional birth) | p-val | <0.001 | <0.001 | <0.001 |  |  |
| Birth interval <24m | coef. | -0.000 | 0.003 | 0.000 | NA | -100.00% |
| (vs >24m) | p-val | (0.987) | (0.130) | (0.648) |  |  |
| 3-4 Children born | coef. | 0.001 | 0.004*** | -0.002* | -300.00% | -150.00% |
| (vs 1-2 children) | p-val | (0.102) | (0.003) | (0.061) |  |  |
| 5+ Children born | coef. | 0.003*** | 0.014*** | -0.004*** | -233.33% | -128.57% |
| (vs 1-2 children) | p-val | (0.002) | <0.001 | <0.001 |  |  |
| Teenage mother (<20 yrs) | coef. | 0.003*** | 0.004** | 0.001 | -66.67% | -75.00% |
| (vs >19 years) | p-val | (0.003) | (0.011) | (0.357) |  |  |
| Mother <145 cm | coef. | 0.009*** | 0.010*** | 0.008*** | -12.50% | -36.36% |
| (vs >155 cm) | p-val | <0.001 | <0.001 | <0.001 |  |  |
| Mother 145-150 cm | coef. | 0.006*** | 0.010*** | 0.003** | -40.00% | -66.67% |
| (vs >155 cm) | p-val | <0.001 | <0.001 | (0.030) |  |  |
| Mother 150-155cm | coef. | 0.001* | 0.002 | 0.001 | 0.00% | -50.00% |
| (vs >155cm) | p-val | (0.087) | (0.239) | (0.195) |  |  |
| Improved latrine | coef. | -0.014*** | -0.020*** | -0.010*** | -28.57% | -50.00% |
| (vs no latrine) | p-val | <0.001 | <0.001 | <0.001 |  |  |
| Unimproved latrine | coef. | -0.010*** | -0.015*** | -0.006*** | -33.33% | -60.00% |
| (vs no latrine) | p-val | <0.001 | <0.001 | <0.001 |  |  |
| Improved water | coef. | 0.003*** | 0.004** | 0.003** | 0.00% | -25.00% |
| (vs unimproved water) | p-val | (0.001) | (0.010) | (0.013) |  |  |
| Rural | coef. | -0.004*** | -0.005*** | -0.004*** | 0.00% | 0.00% |
| (vs urban) | p-val | <0.001 | (0.004) | (0.001) |  |  |
| Boy | coef. | 0.012*** | 0.018*** | 0.007*** | -36.36% | -61.11% |
| (vs girl) | p-val | <0.001 | <0.001 | <0.001 |  |  |
| Father missing | coef. | -0.005*** | -0.004** | -0.003* | -75.00% | -80.00% |
| (vs present) | p-val | <0.001 | (0.041) | (0.051) |  |  |
|  |  |  |  |  |  |  |
| R-squared |  | 0.154 | 0.137 | 0.158 |  |  |

Notes:

*** p<0.01, ** p<0.05, * p<0.1 p-values are based on standard errors adjusted for clustering at the DHS cluster level. Regressions include dummy variables for each of the 125 surveys (country-year fixed effects) and dummies each month of children’s age.

a. These are the percentage differences between the coefficients reported in columns (1) and (3) and (2) and (3), with Wald tests of the null hypothesis of coefficient equality across the regression equations.
