# Supplementary figures and images for: The timing of growth faltering has important implications for observational analyses of the underlying determinants of nutrition outcomes
Source: PLoS One. 2018 Apr 25;13(4):e0195904. doi: 10.1371/journal.pone.0195904 (PMC5919068; doi:10.1371/journal.pone.0195904)

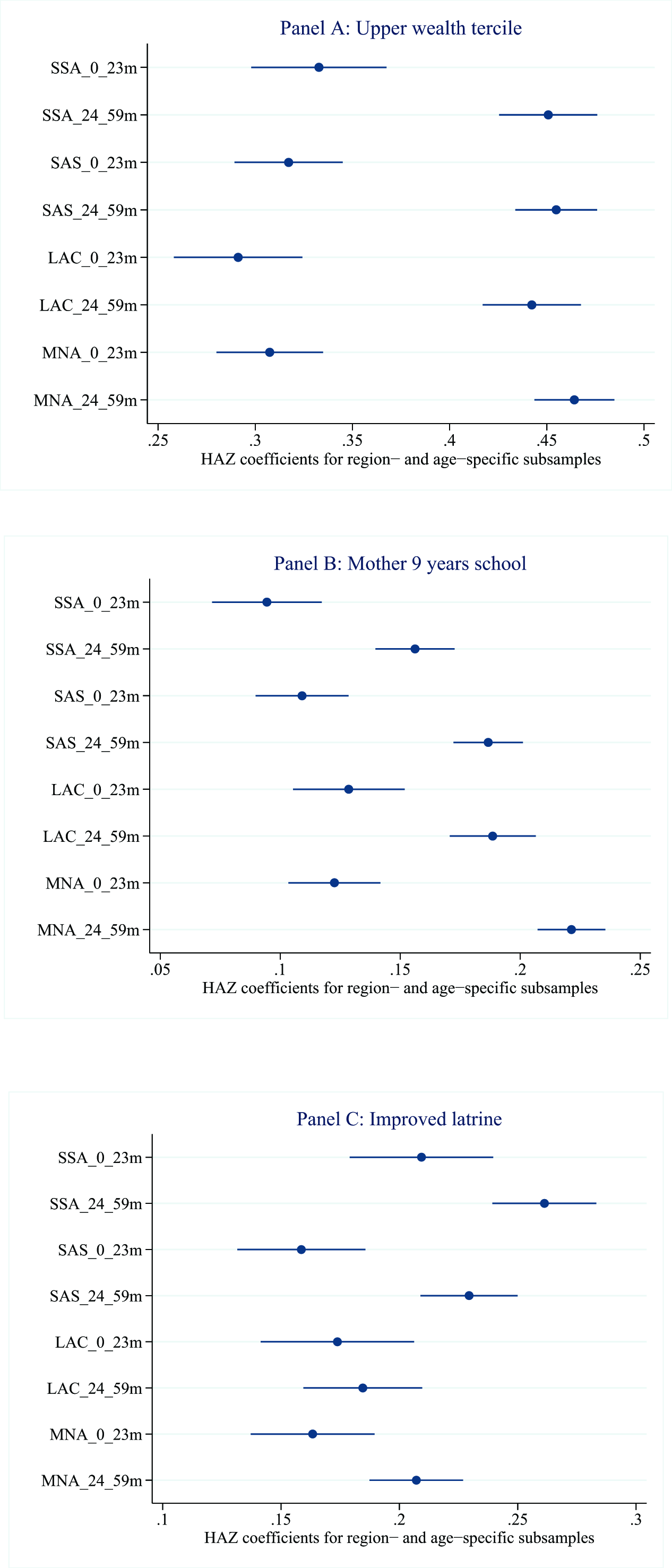

Supplement: S1 Fig — Note: SSA (Sub-Saharan Africa); SAS (South Asia), LAC (Latin America and the Caribbean and MNA (Middle East and North Africa) (TIF) [file pone.0195904.s005.tif]

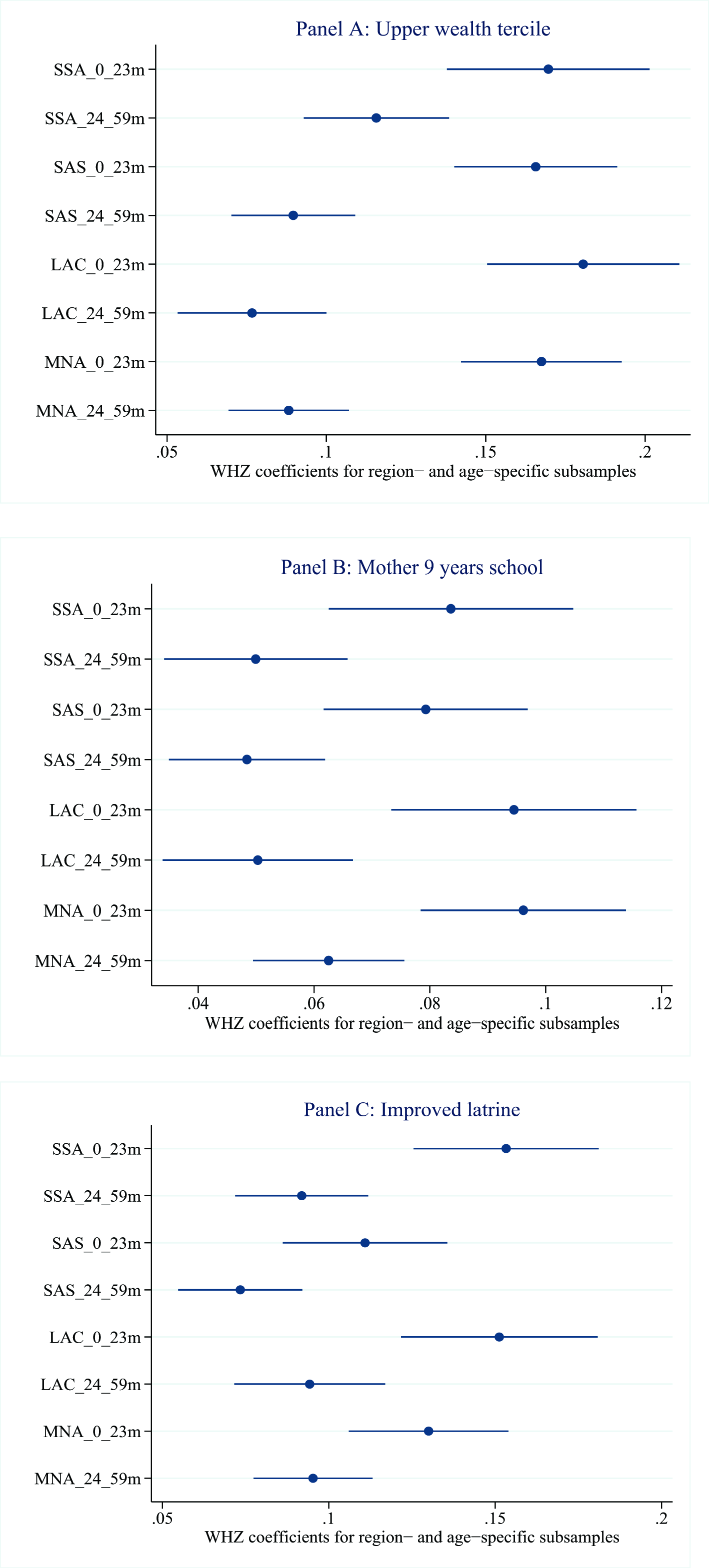

Supplement: S2 Fig — Note: SSA (Sub-Saharan Africa); SAS (South Asia), LAC (Latin America and the Caribbean and MNA (Middle East and North Africa) (TIF) [file pone.0195904.s006.tif]
